# Bcl-2-associated athanogene 5 (BAG5) regulates Parkin-dependent mitophagy and cell death

**DOI:** 10.1038/s41419-019-2132-x

**Published:** 2019-12-02

**Authors:** Mitchell L. De Snoo, Erik L. Friesen, Yu Tong Zhang, Rebecca Earnshaw, Geneviève Dorval, Minesh Kapadia, Darren M. O’Hara, Victoria Agapova, Hien Chau, Ornella Pellerito, Matthew Y. Tang, Xinzhu Wang, Gerold Schmitt-Ulms, Thomas M. Durcan, Edward A. Fon, Lorraine V. Kalia, Suneil K. Kalia

**Affiliations:** 1 Krembil Research Institute, Toronto Western Hospital, University Health Network, 60 Leonard Avenue, Toronto, ON Canada; 2 Department of Laboratory Medicine and Pathobiology, University of Toronto, 1 King’s College Circle, Toronto, ON Canada; 3 McGill Parkinson Program, Department of Neurology & Neurosurgery, Montreal Neurological Institute, McGill University, Montréal, QC Canada; 4 Tanz Centre for Research in Neurodegenerative Diseases, University of Toronto, Toronto, ON Canada; 5 Morton and Gloria Shulman Movement Disorders Clinic and the Edmond J. Safra Program in Parkinson’s Disease, Division of Neurology, Department of Medicine, Toronto Western Hospital, University Health Network, 399 Bathurst Street, Toronto, ON Canada; 6 Division of Neurosurgery, Department of Surgery, University of Toronto, 149 College Street, Toronto, ON Canada

**Keywords:** Mitophagy, Apoptosis, Chaperones, Parkinson's disease

## Abstract

As pathogenic Parkin mutations result in the defective clearance of damaged mitochondria, Parkin-dependent mitophagy is thought to be protective against the dopaminergic neurodegeneration observed in Parkinson’s disease. Recent studies, however, have demonstrated that Parkin can promote cell death in the context of severe mitochondrial damage by degrading the pro-survival Bcl-2 family member, Mcl-1. Therefore, Parkin may act as a ‘switch’ that can shift the balance between protective or pro-death pathways depending on the degree of mitochondrial damage. Here, we report that the Parkin interacting protein, Bcl-2-associated athanogene 5 (BAG5), impairs mitophagy by suppressing Parkin recruitment to damaged mitochondria and reducing the movement of damaged mitochondria into the lysosomes. BAG5 also enhanced Parkin-mediated Mcl-1 degradation and cell death following severe mitochondrial insult. These results suggest that BAG5 may regulate the bi-modal activity of Parkin, promoting cell death by suppressing Parkin-dependent mitophagy and enhancing Parkin-mediated Mcl-1 degradation.

## Introduction

Loss-of-function mutations in the genes encoding Parkin, an E3 ubiquitin ligase, and PINK1, a serine/threonine kinase, cause genetic forms of Parkinson’s disease (PD), a neurodegenerative movement disorder characterized by loss of dopaminergic neurons^1,2^. Parkin and PINK1 act in concert in a recently defined pathway that leads to the specific autophagic degradation of damaged mitochondria, a process known as mitophagy^3^. In this pathway, PINK1 is stabilized on the surface of damaged mitochondria where it phosphorylates its substrates, Parkin and ubiquitin, which results in the recruitment of Parkin from the cytosol to the outer mitochondrial membrane (OMM)^4–7^. Parkin then mediates the formation of polyubiquitin chains on many OMM substrates, including translocase of the outer membrane subunits, voltage-dependent anion channels, and mitofusins, which are subsequently phosphorylated by PINK1^8,9^. This feed-forward process leads to the recruitment of autophagy receptors and engulfment of the damaged mitochondria by autophagosomes^10^. In addition to this wholesale mitophagy, PINK1 and Parkin can initiate the piecewise degradation of mitochondrial components through the generation of mitochondrial derived vesicles (MDVs) containing oxidized electron transport chain components^11^, as well as direct the selective degradation of abnormal protein aggregates from within the mitochondria^12^. Parkin has also been shown to promote the clearance of mitochondria in the absence of the core autophagy proteins by directing endosomes to engulf and transport mitochondria to the lysosomes^13^.

As loss-of-function mutations in either Parkin or PINK1 can result in neurodegeneration, it has been commonly suggested that Parkin and PINK1 function to promote cell survival. However, recent work indicates that, under certain circumstances of mitochondrial stress, Parkin and PINK1 promote apoptosis by directing the polyubiquitination and subsequent degradation of the antiapoptotic Bcl-2 family member, Mcl-1^14,15^. Hence, there is an emerging hypothesis that Parkin may act as a sensor of mitochondrial stress facilitating a switch between pro-survival and pro-death states in cells^14,15^. However, the factors that direct Parkin to function as a promoter of cell death have not yet been fully elucidated.

The Bcl-2 associated athanogene (BAG) family of proteins are co-chaperones known to act in both cell survival and cell death pathways. Each of the six BAG family members (BAG1 to BAG6) contains at least one copy of an evolutionarily conserved domain, the ‘BAG-domain’, that allows them to interact with Hsp70 family molecular chaperones. We previously demonstrated that BAG5 enhances dopaminergic neurodegeneration in rodent models of PD, as well as physically interacts with Parkin^16^. Recently, BAG5 has also been found to interact with PINK1^17^, suggesting a possible role for this co-chaperone in the regulation of the Parkin–PINK1 mitophagy pathway. Two other BAG family members, BAG2 and BAG4, have been shown to differentially modulate recruitment of Parkin to depolarized mitochondria^18,19^ and BAG3 has been identified as a risk locus for PD^20^. Therefore, we sought to determine whether BAG5 regulates Parkin recruitment to mitochondria following mitochondrial depolarization and subsequent mitophagy, and to investigate the role of BAG5 in Parkin-mediated Mcl-1 degradation and cell death.

## Materials and methods

### Cell culture

U2OS cells stably expressing GFP and GFP-Parkin alone, or in combination with mito-mKeima, were generated as we previously described^21,22^. U2OS cells were maintained at 37 °C and 5% CO_2_ in Dulbecco's Modified Eagle Medium (DMEM) (Life Technologies) supplemented with 10% fetal bovine serum (FBS) (Life Technologies), and 1% penicillin/streptomycin (Life Technologies).

Doxycycline inducible GFP and GFP-BAG5 SH-SY5Y cell lines were generated by inserting a Tet-responsive cassette containing either GFP or GFP-BAG5 into the AAVS1 safe harbor, followed by selection in media containing 1.2 μg/mL puromycin for 14 days. SH-SY5Y GFP and GFP-BAG5 cells were maintained at 37 °C and 5% CO_2_ in DMEM (Life Technologies) supplemented with 10% tetracycline-free FBS (Life Technologies), and 1% penicillin/streptomycin (Life Technologies).

### Transfection

For overexpression experiments, cells were transfected with the indicated plasmid according to manufacturer’s protocol for Lipofectamine 2000 (Thermo Fisher Scientific) 24 h prior to treatment. For knockdown experiments, cells were transfected with the indicated siRNA according to manufacturer’s protocol for Lipofectamine RNAiMAX (Thermo Fisher Scientific) 48 h prior to treatment. All siRNAs were purchased from Ambion and include non-targeting control siRNA (siNTC, 4390843) and siRNAs targeting BAG5 (#1 s18285, #2 s18284), PINK1 (s35168), and Parkin (s224170). For experiments involving both knockdown and overexpression, cells were first transfected with siRNA and 24 h later with the indicated expression plasmid.

### CRISPR/Cas-9 Parkin KO cells

Using a CRISPR/Cas-9 approach, as we have previously described^23^, we generated a Parkin knockout (KO) HEK293T cell line. In brief, HEK293T cells were transiently transfected with two gRNA-Cas9 plasmids (px459, Addgene 62988), each encoding a sgRNA-targeting exon one of PARK2 gene;

sgRNA1: CTCCAGCCATGGTTTCCCAG and sgRNA2: CTGCGAAAATCACACGCAAC.

After 48 h, puromycin is added for selection of transfected cells. Clonal cell lines were isolated by serial dilution to isolate single cells per well in a 96 well dish. Each colony was screened for shorter fragment with primers external to exon 1, F:AAGGGCTTCGAGTGATGCTC, R: CCTTGCTGCTCCTGTAGTCA. Confirmation of genetic knockout was confirmed by Sanger sequencing. The wild-type (WT) HEK293T cell line was used as a control for comparison. HEK293T cells were maintained at 37 °C and 5% CO2 in DMEM (Life Technologies) supplemented with 10% FBS (Life Technologies), and 1% penicillin/streptomycin (Life Technologies).

### Fixed cell immunofluorescence microscopy

U2OS GFP-Parkin cells were grown overnight in Nunc Lab-Tek II 8-well chamber slides (Thermo Fisher Scientific) before transfection with dsRed, or FlagBAG5. At 24 h post transfection, cells were treated with 20 μm carbonyl cyanide m-chlorophenyl hydrazone (CCCP) (Sigma Aldrich) for 1 h. Cells were fixed in 4% paraformaldehyde, permeabilized in 0.2% Triton X-100 in phosphate-buffered saline (PBS), and blocked in 5% bovine serum albumin in PBS at room temperature. Cells were incubated overnight at 4 °C with primary antibodies for Flag (M2, Sigma, 1:1000) and TOM20 (Abcam, 1:1000) and then with Alexa Fluor conjugated secondary antibodies (Thermo Fischer Scientific, 1:1000) for 1 h at room temperature. DAPI was incorporated into the final PBS wash at a final concentration of 300 nm. Cells were coverslipped with anti-fade fluorescent mounting medium (Dako). Images were acquired using an LSM 880 laser scanning confocal microscope (Zeiss) using a × 20, 0.75 NA, or × 63, 1.42 NA, lens (Advanced Optical Microscopy Facility at Wright Cell Imaging Facility). GFP-Parkin recruitment to mitochondria was quantified by the visualization of punctate GFP-Parkin that colocalized with the mitochondria marker, TOM20.

### Live cell time-lapse microscopy

U2OS GFP-Parkin cells were plated into 8-well Nunc Lab-Tek II chambered coverglass (Thermo Fisher Scientific) containing siRNA (final concentration 10 nm), Lipofectamine RNAiMAX, and OptiMEM 48 h before imaging. Media was changed 24 h after plating. Cells were infected with CellLight mitochondria-RFP (Thermo Fisher Scientific) 16 h prior to imaging to visualize the mitochondria. Microscopy was performed on a Nikon A1R scanning confocal microscope equipped with an incubator maintained at 37 °C, and 5% CO_2_. Images were acquired using a × 20 0.75 NA CFI Plan Apo VC lens with GFP and RFP excited by 488 nm and 561 nm laser lines, respectively. Fluorescence images were acquired at 5-min intervals for 120 min. For analysis, a minimum of 200 cells per condition were scored by a blinded counter for colocalization between punctate GFP-Parkin and mito-RFP across three independent experiments.

### Mito-mKeima mitophagy assay

U2OS cells stably expressing both GFP-Parkin and ecdysone-inducible mito-mKeima were plated into six-well plates containing siRNA (final concentration 10 nm), Lipofectamine RNAiMAX, and OptiMEM. Media was changed to DMEM containing 10 μm ponasterone A (Sigma) 24 h later to induce expression of mito-mKeima. Cells were treated with 20 μm CCCP for 4 h at 48 h post transfection. For flow cytometry analysis, cells were trypsinized, washed, resuspended in PBS, and analyzed using a LSR Fortessa Cell Analyzer (BD Bioscience). Lysosomal mito-mKeima was detected through a ratiometric pH measurement where pH 7 was detected by excitation with 405 nm laser and pH 4 was detected by excitation with 561 nm laser. A 610/20 emission filter was applied to detect emissions from both excitation lasers. For each sample, 50,000 events were collected, and cells were gated for GFP-Parkin and mito-mKeima expression. Mitophagy was assessed as the percentage of cells displaying an elevated 561/405 nm ratio as captured in the upper gate. This upper gate was defined by a control sample reflecting the baseline mitophagy seen in our system (3–5%), as we have previously described^22,23^. Data were analyzed using FlowJo X.

### Cell viability assay

SH-SY5Y GFP and GFP-BAG5 stable cell lines were treated with 2 μg/mL doxycycline for 24 h to induce transgene expression, and subsequently treated for 18 h with the specified toxin and dose. Cell viability was measured by incubating cells with the PrestoBlue reagent (Thermo Fisher Scientific) for 1 h at 37 °C and analyzing fluorescence on a CLARIOstar plate-reader (BMG Labtech).

### Cell death assay

For BAG5 overexpression experiments, SH-SY5Y GFP and GFP-BAG5 stable cell lines were treated with 2 μg/mL doxycycline for 24 h to induce transgene expression, and subsequently treated for 18 h with 50 μm CCCP. For BAG5 KD experiments, the SH-SY5Y GFP stable cell line was transfected with either siNTC or siBAG5 for 48 h prior to the 18 h incubation with 50 μm CCCP. For Parkin KD experiments, the SH-SY5Y GFP-BAG5 stable cell line was transfected with either siNTC or siParkin for 48 h prior to the 18 h incubation with 50 μm CCCP. For HEK293T cell death assays, HEK293T WT and Parkin KO cells were transfected with either GFP or GFP-BAG5 for 24 h prior to the 24 h incubation with 50 µM CCCP. After CCCP treatment, cells were stained with the DRAQ7 reagent and percentage of DRAQ7 positive cells was assessed by flow cytometry.

### Analysis of PARP and Caspase-3 cleavage and Mcl-1 degradation

SH-SY5Y GFP and GFP-BAG5 stable cell lines were treated with 2 μg/mL doxycycline for 24 h followed by an 18 h incubation with either 1 μm MG132, 50 μm CCCP, or both. The 18 h treatment timepoint was chosen because this was the first timepoint at which CCCP-induced Mcl-1 degradation became visible in the SH-SY5Y stable cell lines. For the experiments containing exogenous Parkin, either myc-tagged Parkin or pcDNA3.1 empty vector was transfected into the cells for 24 h prior to drug treatment using Lipofectamine 2000 as per the manufacturer’s protocol. For the experiments containing BAG5 KD, either siNTC or siBAG5 were transfected into the cells for 48 h prior to drug treatment using Lipofectamine RNAiMAX as per the manufacturer’s protocol. After the drug treatment, poly (ADP-ribose) polymerase (PARP), Caspase-3 and Mcl-1 were assessed via western blot by running whole-cell lysate on 4–15% polyacrylamide gels.

### HA-tag immunoprecipitation

SH-SY5Y cells stably overexpressing GFP or GFP-BAG5 were transfected with either siNTC or siParkin plus HA-Ubiquitin and then treated with 50 μm CCCP for 18 h. Lysates from these cells were incubated overnight at 4 °C with anti-HA-tag antibody conjugated sepharose beads (Cell Signaling Technology 3956). The beads were washed five times in radioimmunoprecipitation assay buffer and hemagglutinin-tagged complexes were eluted by competition with 1 μg/μL synthetic HA peptide (Sino Biological Inc) in tris-buffered saline. Immunoprecipitates were analyzed by subsequent western blot.

### Antibodies

Antibodies used include anti-Flag M2 (Sigma, F3165), anti-TOM20 (Abcam, ab78547), anti-BAG5 (Cusabio, CSB-PA890743HA01HU), anti-Actin (Sigma, A2066), anti-GAPDH (Cell Signaling Technology, 14C10), anti-GFP (Abcam, Ab290), anti-Parkin (Cell Signaling Technology, 2132S), anti-Caspase-3 (Enzo, ADI-AAP-113-D), anti-Cleaved Caspase-3 (Cell Signaling Technology, 9664S), anti-Tubulin (Cell Signaling Technology, 2148S), anti-Mcl-1 (Cell Signaling Technology, 4572S), anti-HA-tag antibody conjugated sepharose beads (Cell Signaling Technology 3956), and anti-HA (Sigma, 11867423001).

### Statistical analyses

Comparisons between groups were performed using two-tailed independent sample student’s *t* tests, or, in the case of more than two groups, one-way analysis of variance (ANOVA) with Bonferroni post hoc testing. Parkin recruitment and cell viability curves were analyzed using two-way ANOVA with Bonferroni or Tukey post hoc testing. All statistical analyses were performed on Prism 7 software (GraphPad).

## Results

### BAG5 delays recruitment of Parkin to depolarized mitochondria

**Fig. 1:**
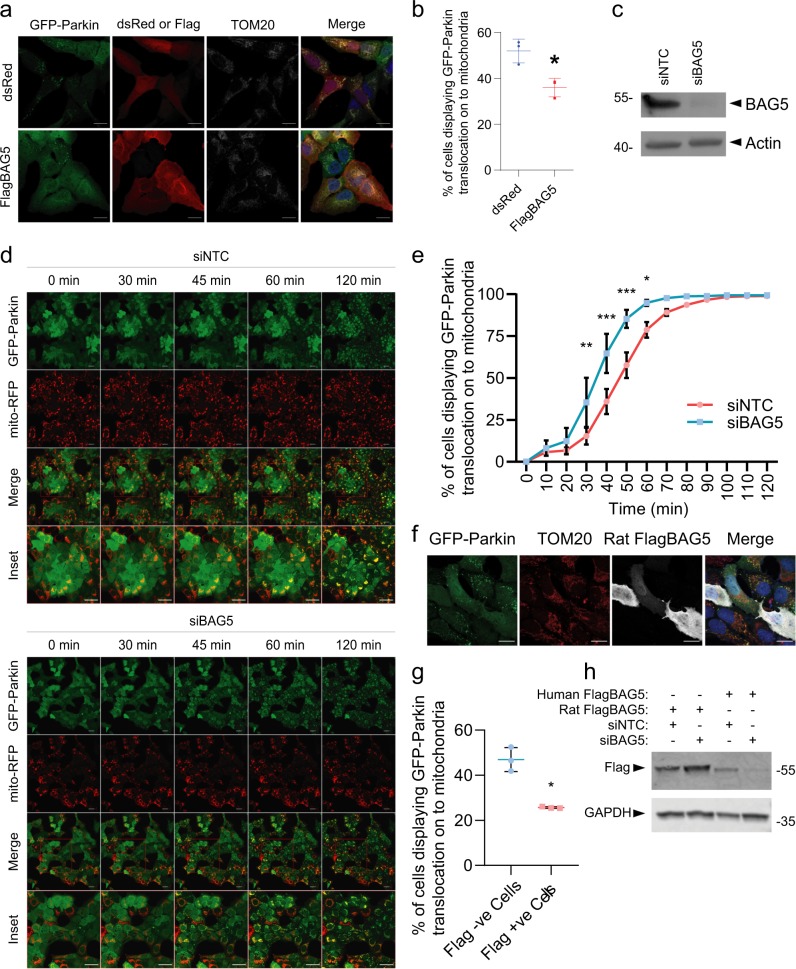
BAG5 regulates Parkin recruitment to mitochondria. **a** Representative confocal micrographs of U2OS GFP-Parkin cells transfected with dsRed or FlagBAG5 and treated with 20 μm CCCP for 60 min. Scale bar is 20 μm. **b** Quantification of the percentage of transfected cells displaying GFP-Parkin recruitment onto the mitochondria by assessing colocalization of GFP-Parkin with TOM20. A minimum of 150 cells per condition were counted in three independent experiments, and statistical significance was determined by student’s *t* test (*p < 0.05). **c** Western blot confirming reduction of endogenous BAG5 protein levels in transfected U2OS GFP-Parkin cells. **d** Representative confocal micrographs of 2 h live cell time-lapse imaging of U2OS GFP-Parkin cells treated with 20 μm CCCP and transfected with non-targeting control or BAG5 siRNA 48 h prior to imaging (see Video 1). **e** Quantification of GFP-Parkin recruitment to mitochondria by evaluating the percentage of cells displaying colocalization between GFP-Parkin and mito-RFP. At least 200 cells were scored per condition by an individual blinded to the treatment. The vertical bars represent the mean ± SEM for three independent experiments. Two-way ANOVA followed by Bonferroni post hoc test revealed siBAG5 transfected cells had a significantly greater percentage of cells displaying GFP-Parkin recruitment to the mitochondria at timepoints between 30 and 60 min inclusive (*p < 0.05, **p < 0.01,***p < 0.001). Scale bar is 50 μm. **f**. Representative confocal micrographs of U2OS GFP-Parkin cells transfected with human BAG5 siRNA and rat FlagBAG5 treated with CCCP for 60 min. Scale bar is 20 μm **g**. Quantification of the percentage of transfected cells displaying GFP-Parkin recruitment onto the mitochondria by assessing colocalization of GFP-Parkin with TOM20. A minimum of 50 Flag negative and Flag positive cells per condition were counted in three independent experiments, and statistical significance was determined by student’s *t* test (*p < 0.05). **h** Representative Western blot demonstrating knockdown of human FlagBAG5 but not rat FlagBAG5 by human BAG5 siRNA.

Given that we have previously shown that BAG5 interacts with Parkin^16^, and that BAG2 and BAG4 have been shown to differentially regulate Parkin recruitment^18,19^, we hypothesized that BAG5 regulates Parkin recruitment to depolarized mitochondria. To test this, we first transfected U2OS cells stably expressing GFP-Parkin with either Flag-tagged BAG5 (FlagBAG5) or dsRed as a control. We then treated the transfected cells with the protonophore, CCCP, which is a well-established method to dissipate the mitochondrial membrane potential and induce GFP-Parkin recruitment to the mitochondria. To quantify Parkin recruitment, we examined the percentage of transfected cells displaying colocalization of punctate GFP-Parkin with the mitochondrial marker, TOM20 (Fig. 1a). With this approach, Parkin recruitment to the mitochondria is considerable but not complete in U2OS cells 60-minutes after treatment with CCCP^21,22^, allowing us to assess the effect of BAG5 overexpression. We found that FlagBAG5-positive cells exhibited a significantly reduced proportion of cells displaying GFP-Parkin colocalization with TOM20 compared with dsRed transfected control cells (dsRed 52.0 ± 3.0% vs FlagBAG5 36.0 ± 2.3%, *n* = 3 experiments with at least 150 cells per condition per experiment, *p* < 0.05), indicating a potential delay in Parkin recruitment by BAG5 (Fig. 1b). Cells transfected with FlagBAG5 or dsRed and treated with DMSO vehicle instead of CCCP displayed no GFP-Parkin recruitment (Supplemental Fig. 1a)

To exclude the possibility that overexpression of BAG5 may indirectly affect Parkin recruitment, we tested if knockdown of endogenous BAG5 with short interfering RNA (siRNA) targeting BAG5 (siBAG5) enhanced Parkin recruitment to the mitochondria upon membrane depolarization in the same cell model. U2OS GFP-Parkin cells were transfected with siBAG5 or with a non-targeting control siRNA (siNTC). Western blotting of these cell lysates demonstrated that siBAG5 reduced endogenous BAG5 protein levels in the U2OS GFP-Parkin cells relative to the siNTC condition (Fig. 1c). We treated the siRNA transfected cells with CCCP 48 h post transfection and performed live cell imaging of GFP-Parkin recruitment to the mitochondria over 2 h at 5-minute intervals (Video 1). Using this approach, which allowed us to examine the kinetics of Parkin recruitment in real-time, we found that siRNA-mediated BAG5 knockdown enhanced the rate of GFP-Parkin translocation to the mitochondria compared with control siNTC (Fig. 1d). Quantification of GFP-Parkin recruitment demonstrated that a significantly higher percentage of siBAG5 transfected cells displayed colocalization of punctate GFP-Parkin and mitochondrial-RFP from 30 to 60 min after CCCP treatment, compared with siNTC transfected cells (Fig. 1e). To establish that this was not the result of off-target effects, we tested another siRNA, which targets BAG5 mRNA in a different region and found that this also significantly enhanced GFP-Parkin recruitment (Supplemental Fig. 1b–d). To further ensure the specificity of our finding, we performed rescue experiments with rat FlagBAG5 (Fig. 1f, g), which is not substantially reduced by the siBAG5 targeting human BAG5 (Fig. 1h), and we demonstrated that the accelerated GFP-Parkin recruitment caused by siBAG5 was rescued in cells expressing rat FlagBAG5 (Fig. 1f, g). Taken together, the results from these experiments indicate that BAG5 is a negative regulator of Parkin translocation to the mitochondria following mitochondrial membrane depolarization.

### BAG5 impairs mitophagy

Given that BAG5 negatively regulates Parkin mitochondrial translocation, we next examined the effects of BAG5 on subsequent mitochondrial turnover by mitophagy. We previously used a quantitative flow cytometry assay that utilizes the localization of a mitochondrially targeted variant of the pH-sensitive fluorescent protein, mKeima (mito-mKeima)^22^. In this assay, the dynamic quantification of mitophagy is measured using the shift in mito-mKeima’s excitation spectrum in response to engulfment of mitochondria into the acidic environment of the lysosome. The ratiometric comparison of fluorescence in response to excitation by 405 nm and 561 nm wavelength light, which represent mito-mKeima at pH 7 and pH 4, respectively, allows for measurement of the proportion of cells undergoing mitophagy. This approach has been extensively validated previously by our group and others, including validation by electron microscopy^22–26^. Although this method cannot be used to differentiate between mitochondria that are directed into lysosomes by canonical wholesale mitophagy^3^ from those that may be transported through alternative pathways involving MDVs^11^ or endosomes^13^, the use of CCCP, PINK1 knockdown and a catalytically dead Parkin mutant allows for a specific evaluation of PINK1-Parkin-dependent mechanisms of mitophagy in our system.

**Fig. 2:**
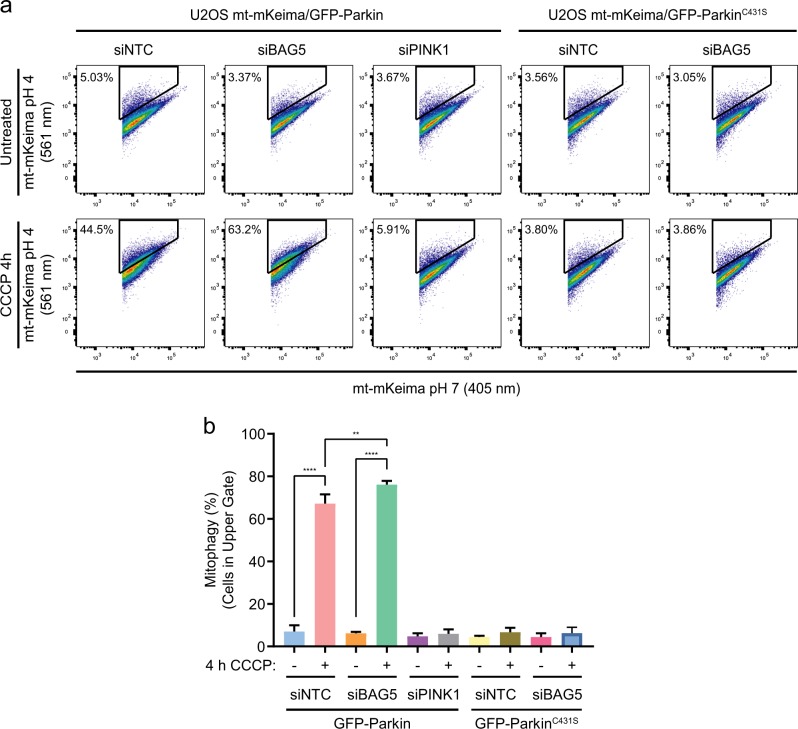
BAG5 knockdown enhances mitophagy. **a** Representative flow cytometry data of U2OS mito-mKeima cell lines also expressing GFP-Parkin or GFP-Parkin^C431S^ that were untreated or treated with 20 μm CCCP for 4 h after transfection with non-targeting, BAG5, or PINK1 siRNA. Mitophagy was assessed as the percentage of cells displaying an elevated 561/405 nm ratio as captured in the upper gate as we have previously described^22,23^. **b** Quantification of average percentage of mitophagy from three independent experiments. Statistical significance was determined by a one-way ANOVA with Tukey’s post hoc test (**p < 0.005, ****p < 0.0001).

U2OS cells stably expressing inducible mito-mKeima, as well as GFP-Parkin, or the catalytically dead mutant, GFP-Parkin^C431S^, were treated with CCCP for 4 h to allow for mitophagy to occur following transfection with siNTC, siBAG5, or siPINK1. BAG5 knockdown significantly enhanced the proportion of cells undergoing mitophagy compared to siNTC (Fig. 2a, b) in cells expressing GFP-Parkin. However, BAG5 knockdown did not enhance the proportion of cells undergoing mitophagy in cells expressing GFP-Parkin^C431S^, indicating that this effect is dependent on Parkin E3 ligase activity. It has been suggested that non-specific acidification of the cytosol by protonophores, rather than depolarization of the mitochondrial membrane, regulates mitophagy^27^. In our system, however, we show that this is not the case as siRNA-mediated knockdown of PINK1 prevents mito-mKeima from becoming acidified following CCCP treatment (Fig. 2a, b), indicating that mito-mKeima becomes engulfed in lysosomes in response to the PINK1-dependent mitophagy pathway rather than a non-specific acidification in the cytosol. Thus, these data demonstrate that, consistent with its inhibition of Parkin recruitment, BAG5 also inhibits PINK1 and Parkin-dependent mitophagy.

### BAG5 enhances CCCP-induced apoptosis

**Fig. 3:**
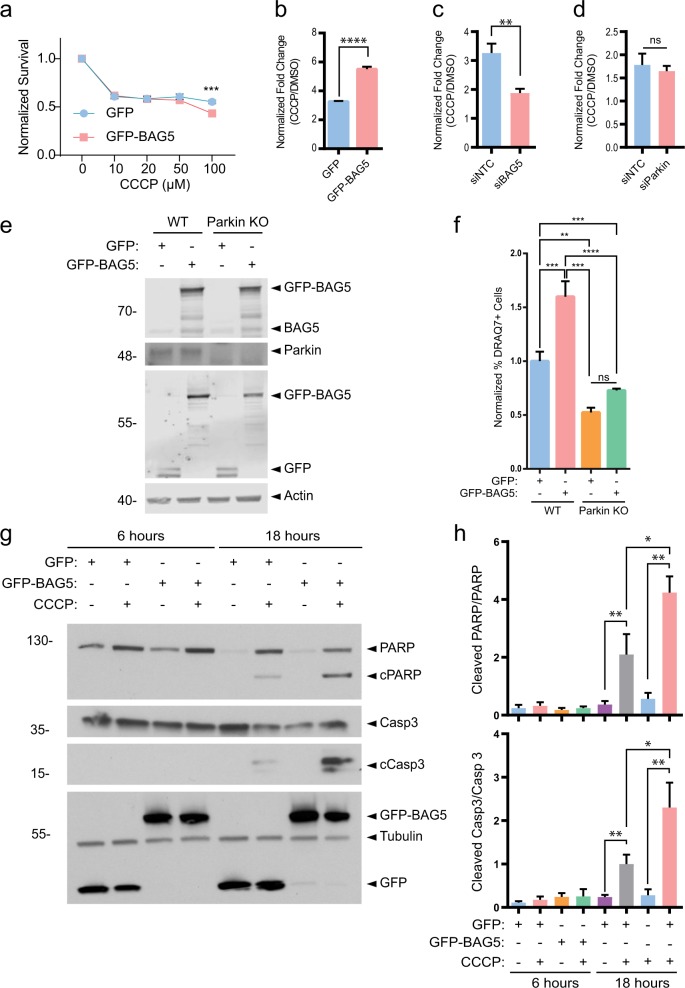
BAG5 enhances cell death and causes increased PARP and Caspase-3 cleavage following CCCP treatment. **a** Dose–response curve for CCCP in the GFP and GFP-BAG5 stable cell lines using PrestoBlue viability assay. Experiment performed three times in triplicate. Data back normalized to 0 treatment condition, and statistical analysis was done using two-way ANOVA followed by Bonferroni post hoc testing (***p < 0.001). **b** Fold change in cell death elicited by 50 μm CCCP treatment (CCCP/DMSO) in the GFP and GFP-BAG5 SH-SY5Y stable cell lines as measured by the DRAQ7 cell death assay using flow cytometry. GFP and GFP-BAG5 expression induced for 24 h using 2 μg/mL doxycycline prior to treatment. Experiment performed two times in triplicate. Statistical significance determined by unpaired *t* test (****p < 0.0001). Columns represent mean ± SEM. **c** Same as **b** comparing the effect of BAG5 KD (siBAG5) to control (siNTC). **d** Same as **b** comparing the effect of Parkin KD (siParkin) to control (siNTC) in SH-SY5Y GFP-BAG5 cells. **e** Western blot demonstrating Parkin knockout (KO) in Parkin KO HEK293T cells compared with the wild-type (WT) HEK293T cells and relative levels of transfected GFP and GFP-BAG5. **f** Relative percentage of cell death elicited by 50 μm CCCP in Parkin KO HEK293T cells relative to WT HEK293T cells transfected with GFP-BAG5 or GFP alone as a control. Experiment performed two times in triplicate. Statistical analysis was done using one-way ANOVA with Tukey’s post hoc test (**p < 0.01, ***p < 0.001, ****p < 0.0001). Columns represent mean ± SEM. **g** Western blot illustrating the changes in endogenous PARP, cleaved PARP (cPARP), Caspase-3 (Casp3), and cleaved Caspase-3 (cCasp3) in the GFP and GFP-BAG5 SH-SY5Y stable cell lines following treatment with 50 μm CCCP. Representative blot from three independent experiments. **h** Quantification of the western blot presented in **g**. Data obtained from three independent experiments and statistical analysis done using one-way ANOVA followed by Bonferroni post hoc testing (*p < 0.05, **p < 0.01, ***p < 0.001, ****p < 0.0001). Columns represent mean ± SEM.

Parkin, when activated by PINK1 in response to mitochondrial depolarization by CCCP, has recently been shown to function as a stress sensor which may direct cells toward apoptosis in response to mitochondrial impairment^14,15^. Given that we previously demonstrated that BAG5 inhibits Parkin E3 ligase activity^16^ and we now show that BAG5 modulates Parkin recruitment and mitophagy following CCCP treatment, we hypothesized that BAG5 also regulates the proapoptotic activity of Parkin associated with mitochondrial depolarization. To test this possibility, we utilized stable SH-SY5Y cell lines with tet-inducible GFP or GFP-BAG5 expression (Supplemental Fig. 2a–d). Unlike U2OS cells, SH-SY5Y cells express detectable levels of endogenous Parkin and the addition of exogenous Parkin expression by transfection enhances cell death in response to mitochondrial damage^14^. Using a resazurin-based viability assay, we found that induction of GFP-BAG5 expression reduced cell viability following CCCP treatment in the SH-SY5Y cell lines (Fig. 3a) as well as in the context of other inducers of cell death (Supplemental Fig. 2e). Resazurin-based viability assays use mitochondrial metabolic activity as a surrogate for cell viability, which may limit their suitability for toxins that directly impact mitochondrial function, such as CCCP. Therefore, we also used a complimentary assay in which cell death is measured by DRAQ7 and, consistent with the resazurin-based viability assay results, we observed that GFP-BAG5 overexpression enhanced CCCP-mediated cell death (Fig. 3b). Conversely, siRNA-mediated knockdown of BAG5 reduced CCCP-mediated cell death (Fig. 3c). However, knockdown of Parkin by siRNA was not sufficient to significantly rescue CCCP-mediated cell death in GFP-BAG5 overexpressing SH-SY5Y cells at this timepoint as measured by DRAQ7 (Fig. 3d). Given the possibility that this may be due to insufficient or transient knockdown of Parkin we used a CRISPR/Cas9 approach^23^ to generate Parkin knockout (KO) HEK293T cells. Genetic disruption was confirmed by sequencing and subsequently by assessing protein expression (Fig. 3e). As we observed in the SH-SY5Y cell lines, GFP-BAG5 overexpression significantly enhanced CCCP-mediated cell death in wild-type (WT) cells (Fig. 3f). Parkin KO resulted in significantly decreased CCCP-mediated cell death and importantly, overexpression of GFP-BAG5 did not enhance cell death in Parkin KO cells as it did in WT cells (Fig. 3f). Taken together this suggests that BAG5 likely acts upstream from Parkin in modulating cell death following mitochondrial depolarization.

As cell death associated with mitochondrial depolarization and Parkin is apoptotic^14,15^, we postulated that the enhanced cell death caused by BAG5 in response to mitochondrial stress also occurs via apoptosis. To test this, we measured caspase-3 cleavage and poly (ADP-ribose) polymerase (PARP) cleavage^28^, both markers of apoptosis. Western blot analysis of the SH-SY5Y cells at 6 h and 18 h following CCCP treatment revealed that GFP-BAG5 enhanced the cleavage of both caspase-3 and PARP following 18 h of toxic mitochondrial depolarization relative to control cell lines that express GFP alone (Fig. 3g, h). Therefore, the activation of the caspase-3 apoptotic pathway following CCCP treatment is enhanced by BAG5, and in turn, promotes CCCP-induced cell death.

### BAG5 modulates the effects of Parkin on Mcl-1 stability

**Fig. 4:**
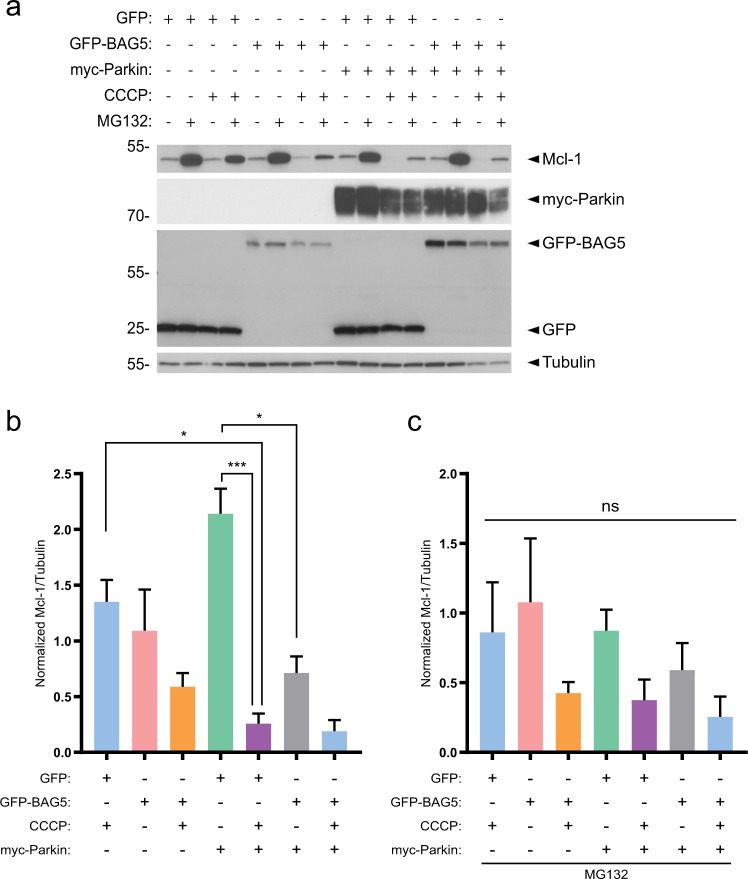
Parkin and BAG5 modulate Mcl-1 degradation following CCCP treatment. **a** Representative western blot illustrating the changes in endogenous Mcl-1 in the GFP and GFP-BAG5 SH-SY5Y cell lines transfected with either pcDNA3.1 vector control or myc-Parkin, and treated with either 1 μm MG132, 50 μm CCCP, or a combination of the two. Representative image obtained from three independent experiments. **b** Quantification of Mcl-1 from western blots in three independent experiments in the absence of MG132. Data were obtained from three independent experiments and normalized to the GFP alone control condition in each experiment. **c** Quantification of Mcl-1 from western blots in independent experiments in the presence of MG132. Data were obtained from three independent experiments and normalized to the GFP alone control condition in each experiment. Statistical analysis in **b** and **c** was done using one-way ANOVA followed by Bonferroni post hoc testing (*p < 0.05, **p < 0.01, ***p < 0.001). Columns represent mean ± SEM.

Parkin enhances apoptosis following CCCP-induced mitochondrial depolarization, in part, via the proteasomal degradation of the pro-survival Bcl-2 family member, Mcl-1^14,15^. As BAG5 also enhanced CCCP-induced apoptosis, we next assessed the effect of BAG5 on Parkin-mediated changes in Mcl-1 stability. Consistent with previous reports^14,15^, we found the presence of exogenous myc-Parkin significantly enhanced Mcl-1 degradation following CCCP treatment (Fig. 4a, b). Less Parkin-mediated Mcl-1 degradation occurred in the presence of the proteasome inhibitor, MG132 (Fig. 4c), supporting previous findings that Parkin promotes the degradation of Mcl-1 by the ubiquitin-proteasome system. MG132 did not completely block Parkin-mediated Mcl-1 degradation, potentially owing to the lower concentration of MG132 (1 μm) used in this study relative to previous reports^14,15^. The lower concentration was necessary due to the significant toxicity associated with MG132 treatment in these stable SH-SY5Y cell lines (Supplemental Fig. 2e). We noted that GFP-BAG5 expression alone did not significantly affect the levels of Mcl-1 in the absence of mitochondrial depolarization by CCCP, compared with GFP expression in the presence or absence of MG132. When combined with expression of exogenous myc-Parkin, GFP-BAG5 expression resulted in a reduction in Mcl-1 levels compared with vehicle-treated GFP cells; however, GFP-BAG5 expression did not have any additional effect on Parkin-mediated Mcl-1 degradation following CCCP treatment (Fig. 4a, b).

**Fig. 5:**
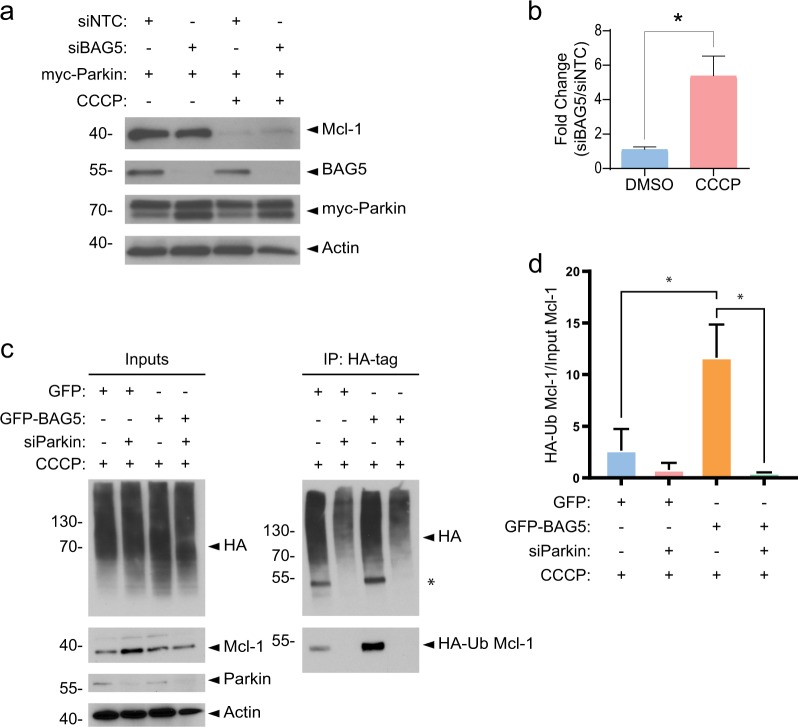
BAG5 knockdown partially rescues Mcl-1 degradation. **a** Western blot illustrating the changes in endogenous Mcl-1, BAG5, and exogenous myc-Parkin elicited by siRNA-mediated BAG5 knockdown (siBAG5) vs. Control (siNTC) with or without CCCP treatment in SH-SY5Y cells. **b** Quantification of Mcl-1 presence in the western blot. Mcl-1 intensity normalized to Actin and expressed as a ratio of the intensity found in the siBAG5 lane vs. siNTC lane. Data obtained from three independent experiments, normalized to siNTC condition and compared using an unpaired *t* test (*p < 0.05). Columns represent mean ± SEM. **c** SH-SY5Y cells overexpressing GFP-BAG5 or GFP were transfected with HA-ubiquitin plus siRNA targeting Parkin (siParkin) or control siRNA and then treated with 50 μm CCCP for 18 h. Ubiquitinated proteins were immunoprecipitated with anti-HA antibodies and analyzed by western blot. *Indicates previously probed band from panel below. **d** Quantification of the level of immunoprecipitated HA-ubiquitinated Mcl-1 (HA-Ub Mcl-1) relative to Mcl-1 protein levels in the inputs. Data obtained from three independent experiments and statistical analysis was done using one-way ANOVA followed by Tukey post hoc testing. Columns represent mean ± SEM.

The degradation of Mcl-1 in the presence of exogenous myc-Parkin was near complete; thus, it is possible that any additional effects of exogenous BAG5 expression on Mcl-1 degradation could not be detected. Therefore, we used siRNA-mediated knockdown of endogenous BAG5 in combination with myc-Parkin overexpression to test if BAG5 may modulate Parkin-mediated degradation of Mcl-1 in response to mitochondrial impairment. Using this approach, we found that BAG5 knockdown reduced Parkin-dependent degradation of endogenous Mcl-1 following mitochondrial depolarization by CCCP (Figure 5a, b). Endogenous BAG5 protein levels did not change following CCCP treatment (Fig. 5a). To understand whether this may occur by BAG5-modulating endogenous Parkin-mediated Mcl-1 ubiquitination, we next performed HA-tag immunoprecipitations from GFP-BAG5 or control GFP-overexpressing SH-SY5Y cells transfected with HA-ubiquitin and treated with CCCP. We found significantly higher levels of HA-conjugated Mcl-1 immunoprecipitated from lysate containing GFP-BAG5 compared with GFP lysate. Furthermore, this effect of enhanced immunoprecipitation of HA-conjugated Mcl-1 was mitigated by Parkin knockdown (Fig. 5c, d). Taken together, these findings suggest that BAG5 enhances both Parkin-mediated Mcl-1 ubiquitination and degradation following mitochondrial impairment and thus may contribute to the enhanced apoptosis associated with Parkin in the context of mitochondrial stress.

## Discussion

Here we demonstrate a new functional relationship between BAG5 and Parkin, which occurs in response to mitochondrial depolarization. We show that BAG5 delays Parkin recruitment to mitochondria following mitochondrial depolarization and subsequently impairs mitophagy. Furthermore, we demonstrate that BAG5 enhances cell death following mitochondrial depolarization and promotes Parkin-mediated Mcl-1 degradation. Together, these results reveal a role for BAG5 in modulating Parkin’s function in mitophagy and cell death.

Our findings that BAG5 inhibits Parkin recruitment to depolarized mitochondria, together with previous reports that BAG2 and BAG4 affect Parkin recruitment^18,19^, suggest a more general role for BAG family proteins in regulation of Parkin recruitment. In parallel to our findings, Tan et al^29^. have found that BAG5 also attenuates mitochondrial Parkin localization in the context of mitochondrial Hexokinase-II dissociation induced-mitophagy, a pathway that may be important in conferring cardioprotection against ischemia. This suggests that BAG5 may play a central role in Parkin recruitment in different types of cell stress^29^. However, it is not yet clear the mechanism by which this regulation may occur as BAG5, like BAG4, impedes Parkin recruitment^19^, whereas BAG2 enhances Parkin recruitment in the context of mitochondrial impairment^18^. The differences between the effects of BAG5 and BAG4 compared with BAG2 may be a result of different interactions between Parkin and/or other proteins mediated by the shortened BAG domains of BAG5 and BAG4^30^ compared with BAG2′s structurally unique C-terminal brand new BAG (BNB) domain^31^. Indeed, both BAG4 and BAG5 interact with Parkin^16,19^. An interaction between BAG2 and Parkin has not yet been demonstrated, but BAG2 is known to interact with PINK1^18^ and inhibit the E3 ligase CHIP^32^, both proteins that interact with Parkin^33^. The apparent opposing functions between BAG family members is not unprecedented as BAG1 and BAG3 are known to oppose each other in an age and stress dependent switch that modulates protein quality control^34^. In this switch, greater expression of BAG1 normally promotes protein turnover through proteasomal degradation but, with age or cellular stress, BAG1 expression decreases, whereas BAG3 expression increases, which promotes a shift to protein turnover by autophagy^34^. It will be important in future studies to investigate if expression patterns of BAG5, BAG4, and BAG2 change with age, potentially constituting a similar functional switch in mitochondrial quality control by differentially regulating Parkin. Consistent with this idea, BAG2 was shown to protect primary cortical neurons from Complex I inhibition^18^, whereas we have shown here that BAG5 enhances apoptosis following mitochondrial depolarization. Another important consideration is that BAG family proteins may have important functions in parallel to BAG5 in proteasome and autophagy lysosome degradation pathways^35^. For instance, BAG3 may be induced by proteasome inhibition and this may in turn modulate Mcl-1 degradation^36,37^. Understanding if Mcl-1 levels and cell survival are modulated uniquely by BAG5 or if other BAG proteins can have synergistic or opposing effects will be an important consideration for future investigation. Adding further complexity in dissecting how exactly BAG family proteins regulate mitophagy is the potential for BAG domains to homodimerize^38^. The functional consequences of this dimerization in the context of Parkin recruitment are not yet understood but it is possible that, in addition to regulating Parkin, BAG family members may be able to regulate each other.

**Fig. 6:**
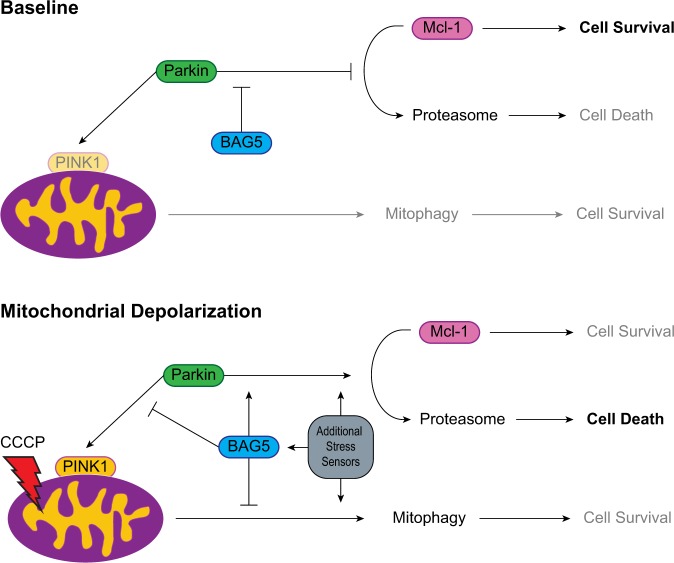
Proposed model for the role of BAG5 in regulating Parkin’s functions in mitophagy and cell death. At baseline conditions, Parkin exists in an inactive state and when activated by PINK1 following mitochondrial depolarization, BAG5 inhibits its recruitment and action in mitophagy while enhancing degradation of Mcl-1, which in turn promotes apoptosis. An additional network of “stress sensor” chaperone proteins is likely required for full activation of Parkin’s cytotoxic action. For example BAG2^18^, BAG4^19^, and CHIP^33^ are co-chaperones involved in context-dependent regulation of PINK1 and Parkin.

Previous reports suggested that Parkin may act as a ‘switch’ or ‘damage-sensor’ by moving between two functional states: a cytoprotective state where Parkin promotes cell survival by sequestering damaged mitochondria to the lysosomes, and a cytotoxic state where Parkin promotes the activation of apoptotic pathways by enhancing the proteasomal degradation of Mcl-1^14,15^. These studies postulated that Parkin is in its cytoprotective state during physiological or mildly stressful conditions, but switches to its cytotoxic state in the face of severe mitochondrial damage. The hypothesis that Parkin can exist in two functional states resembles the known function of p53, which can either promote cellular health by promoting DNA damage repair prior to cell division or activate apoptotic pathways in the case of irreparable damage^39^. Here, we show that BAG5 simultaneously inhibited Parkin-dependent mitophagy and enhanced Parkin-mediated Mcl-1 degradation following CCCP treatment. Thus, our results suggest that BAG5, which we previously demonstrated to be induced following dopaminergic neuron stress^16^, enhances the switching of Parkin to a pro-death pathway (Fig. 6). However, we did not observe an increase in BAG5 protein levels following CCCP treatment, suggesting that the effects of BAG5 on Parkin might be mediated by post-translational modifications or changes in the BAG5 interactome following mitochondrial stress. Our results demonstrate that BAG5 knockdown was capable of partially rescuing Parkin-mediated Mcl-1 degradation following CCCP treatment. Although siRNA-mediated knockdown of Parkin was insufficient to prevent the enhanced cell death observed with BAG5 overexpression, knockout of Parkin using CRISPR/Cas was sufficient to occlude the effect of BAG5 overexpression. Technical differences aside, this suggests the possibility that other proteins may also facilitate the cytotoxic effects of Parkin and/or there exists parallel compensatory pathways, which may also be regulated by BAG5 and other chaperone proteins in the context of severe mitochondrial damage. It will be important to uncover the other members of this regulatory protein network that function alongside BAG5 to provide insight into the relevant molecular pathways responsible for mitochondrial stress detection and switching Parkin between its cytoprotective and cytotoxic states.

Two non-mutually exclusive mechanisms may account for how BAG5 directs Parkin to enhance degradation of mitochondrial Mcl-1 while slowing Parkin recruitment to the mitochondria. First, BAG5 may modulate Parkin’s specificity and/or E3 activity for its substrates culminating in the increased likelihood of ubiquitinating mitochondrial Mcl-1 despite a lower Parkin load on the mitochondria. This is supported by the increased Parkin-dependent HA-ubiquitination of Mcl-1 in the presence of BAG5 and is reminiscent of BAG5's ability to modulate the ubiquitination activity of another E3 ubiquitin ligase, CHIP^40^. Second, Mcl-1 functions to prevent excessive accumulation of the proapoptotic, pore-forming Bcl-2 family members, Bax and Bak, on the outer mitochondria membrane by associating with them and mediating retrotranslocation to the cytosol^41,42^. This retrotranslocated pool of cytosolic Mcl-1 may then be targeted by cytosolic Parkin. Although Parkin is known to ubiquitinate non-mitochondrial substrates PARIS^43^ and intracellular pathogens^44^, how it becomes activated in the cytosol without PINK1 is unclear. Future investigation into mechanisms by which Parkin’s substrate specificity may change in response to cellular stress and how this may be modulated by chaperone proteins such as BAG5 will be essential to understanding its cytotoxic capacity.

In addition to characterizing the functional relationship between BAG5 and Parkin, our results are important for enhancing the understanding of BAG5, which has a largely unknown function despite being implicated in the molecular pathogenesis of both PD and cancer^35,45–47^. This may help to clarify the seemingly contradictory results surrounding the possible involvement of BAG5 in cell death. Some groups have recently reported that BAG5 promotes cell survival^17,46,48–50^, whereas others report that BAG5 enhances cell death^16,51^. In our system, BAG5 promoted apoptosis following exposure to CCCP, but had opposing modulatory effects on cell viability in the context of other toxins. This fits with the notion that BAG5 acts as sensor within ‘switching’ cellular pathways that serve to promote cell survival in some contexts and death in others. BAG5 was recently shown to functionally interact with p53^47^ and it is intriguing to speculate that BAG5 may act as a more global regulator of proteins that mediate context-dependent effects on cell death pathways. It is possible that the findings in the literature are not in disagreement with each other, but rather illustrate the context-dependent effects of BAG5, like Parkin, on apoptosis. Therefore, caution should be taken in labeling BAG5, and potentially other BAG proteins, as either ‘pro-survival’ or ‘proapoptotic’, as it is likely to be an oversimplification of a reality that involves a network of proteins with complex and opposing effects on modulating cell death in specific paradigms.

## Supplementary information

Video

Supplemental Figure 1

Supplemental Figure 2

Supplementary figures
