# Supplementary figures and images for: Bcl-2-associated athanogene 5 (BAG5) regulates Parkin-dependent mitophagy and cell death
Source: Cell Death Dis. 2019 Dec 2;10(12):907. doi: 10.1038/s41419-019-2132-x (PMC6885512; doi:10.1038/s41419-019-2132-x)

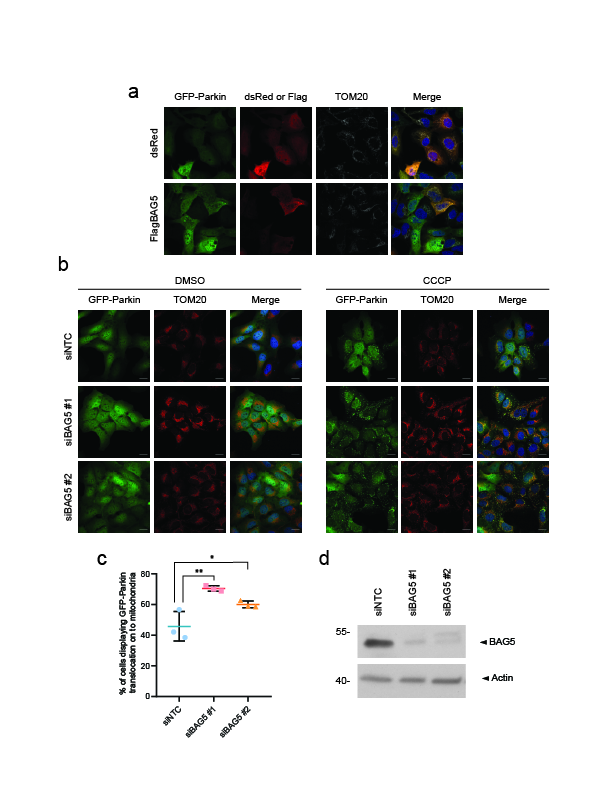

Supplement: Supplementary file 2 — Supplemental Figure 1 [file 41419_2019_2132_MOESM2_ESM.tif]

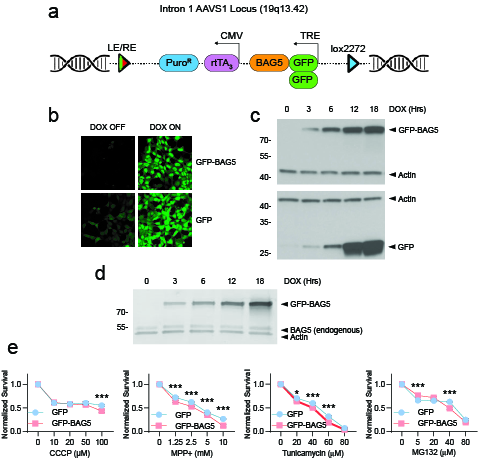

Supplement: Supplementary file 3 — Supplemental Figure 2 [file 41419_2019_2132_MOESM3_ESM.tif]
