# Supplementary material for: Bcl-2-associated athanogene 5 (BAG5) regulates Parkin-dependent mitophagy and cell death
Source: Cell Death Dis. 2019 Dec 2;10(12):907. doi: 10.1038/s41419-019-2132-x (PMC6885512; doi:10.1038/s41419-019-2132-x)
Supplement: Supplementary file 4 — Supplementary figures [file 41419_2019_2132_MOESM4_ESM.docx]

**Supplemental Figure 1: BAG5 overexpression alone does not enhance parkin recruitment without mitochondrial depolarization whereas multiple BAG5 siRNAs enhance GFP-Parkin recruitment to depolarized mitochondria. a.** Representative confocal micrographs of U2OS GFP-Parkin cells transfected with dsRed or FlagBAG5 and treated with DMSO for 60 min. Scale bar is 20 μm. **b.** Representative confocal micrographs of U2OS GFP-Parkin transfected with non-targeting control (siNTC) or two unique siRNA targeting human BAG5 mRNA (siBAG5 #1 and siBAG5 #2) and treated with 20 μM CCCP or DMSO vehicle for 1 hour. Scale bar is 20 μM **c.** Quantification of the percentage of transfected cells displaying GFP-Parkin recruitment onto the mitochondria by assessing colocalization of GFP-Parkin with TOM20. A minimum of 250 cells per condition were counted in three independent experiments, and statistical significance was determined by one-way ANOVA and Bonferroni post-hoc testing (*p<0.05,**p<0.01). **d.** Representative western blot demonstrating knockdown efficiency of BAG5 siRNAs.

**Supplemental Figure 2:** **Generation of SH-SY5Y stable cell lines reveals that BAG5 has a modulatory effect on toxin-induced reductions in cell viability**. **a.** Schematic of the strategy used to insert the GFP and GFP-BAG5 transgenes into the AAVS1 safe harbour of SH-SY5Y cells. **b.** Representative confocal images of the induction of the GFP transgenes following an 18 h incubation with doxycycline (DOX ON) vs. DMSO control (DOX OFF). **c.** Western blot of SH-SY5Y cell lysate probed with anti-GFP antibody after the GFP and GFP-BAG5 transgenes were induced for the indicated time with doxycycline (DOX). **d.** Western blot of the SH-SY5Y cell lysate probed with anti-BAG5 antibody after the GFP-BAG5 transgene was induced for the indicated time with doxycycline (DOX) **e.** Panel of viability assays conducted using the PrestoBlue viability assay. Data back normalized to 0 treatment condition, and statistical analysis was done using 2-way ANOVA followed by Bonferroni post-hoc testing (*p<0.05, **p<0.01, ***p<0.001, ****p<0.0001).

**Video 1: BAG5 siRNA accelerates GFP-Parkin translocation.** Live cell 2 h time-lapse imaging comparing GFP-Parkin translocation in U2OS GFP-Parkin cells transfected with non-targeting (siNTC) or BAG5 siRNA (siBAG5) for 48 h before treatment with 20 μM CCCP.
